# Evaluation of an intensive education program on the treatment of tobacco-use disorder for pharmacists: a study protocol for a randomized controlled trial

**DOI:** 10.1186/s13063-018-3068-7

**Published:** 2019-01-08

**Authors:** Maguy Saffouh El Hajj, Ahmed Awaisu, Nadir Kheir, Mohamad Haniki Nik Mohamed, Rula Shami Haddad, Rana Ahmed Saleh, Noora Mohammed Alhamad, Ahmad Mohd Almulla, Ziyad R. Mahfoud

**Affiliations:** 1 College of Pharmacy, Qatar University, Doha, 2713 Qatar; 2 School of Pharmacy, Faculty of Medical and Health Sciences, University of Auckland, Auckland, New Zealand; 3 Kulliyyah of Pharmacy, International Islamic University Malaysia, 25200 Kuantan, Pahang Malaysia; 4 Tobacco Control Center-WHO Collaborating Center, Hamad Medical Corporation, Doha, Qatar; 5 Weil Cornell Medicine-Qatar, PO Box 24144, Doha, Qatar

**Keywords:** Qatar, Education program, Tobacco control, Smoking cessation, Pharmacist

## Abstract

**Background:**

Tobacco use is presently responsible for the death of over seven million people across the world. In Qatar, it is one of the main causes of premature deaths and preventable diseases. To reduce tobacco use, Qatar has ratified the World Health Organization (WHO)’s Framework Convention on Tobacco Control (FCTC) and has implemented many tobacco-control initiatives. In spite of these measures, tobacco use is still considered a public health threat in Qatar. Pharmacists practicing in retail/community pharmacy settings are often the first port of call for individuals requiring general health advice. Evidence has proven that they have a pivotal role in health promotion and disease prevention including tobacco cessation. However, pharmacists in Qatar are not actively involved in tobacco control and many have not received any education or training about smoking cessation counseling in the past. In an effort to build the capacity of pharmacists towards tobacco control in Qatar, the aim of the proposed study is to design, implement, and evaluate an intensive education program on tobacco dependence treatment for pharmacists in Qatar.

**Methods/design:**

The study will be a prospective randomized controlled trial comparing an intensive tobacco-related education program versus non-tobacco-related training on pharmacists’ tobacco-use-related knowledge, attitudes, self-efficacy, and skills. Community pharmacists practicing in Qatar will be eligible for participation in the study. A random sample of pharmacists will be selected for participation. Consenting participants will be randomly allocated to intervention or control groups. Participants in the intervention group will receive an intensive education program delivered by a multi-disciplinary group of educators, researchers, and clinicians with expertise in tobacco cessation. A short didactic session on a non-tobacco-related topic will be delivered to pharmacists in the control group. The study has two primary outcomes: post-intervention tobacco-related knowledge and post-intervention skills for tobacco cessation assessed using a multiple-choice-based evaluation instrument and an Objective Structured Clinical Examination (OSCE), respectively. The secondary study outcomes are post-intervention attitudes towards tobacco cessation and self-efficacy in tobacco-cessation interventions assessed using a survey instrument. An additional secondary study outcome is the post-intervention performance difference in relation to tobacco-cessation skills in the practice setting assessed using the simulated client approach.

**Discussion:**

If demonstrated to be effective, this education program will be considered as a model that Qatar and the Middle East region can apply to overcome the burden of tobacco-use disorder.

**Trial registration:**

ClinicalTrials.gov, ID: NCT03518476. Registered on 8 May 2018. Version 1/22 June 2018.

**Electronic supplementary material:**

The online version of this article (10.1186/s13063-018-3068-7) contains supplementary material, which is available to authorized users.

## Background

Tobacco use is one of the leading causes of premature morbidity and mortality in the world causing over seven million deaths a year [1]. With the current trend, it is projected that tobacco use will cause more than eight million deaths per year by 2030 [2]. Tobacco-use cessation entails many short- and long-term health benefits for the user, the healthcare system, and to society [3]. These benefits include, but are not limited to, reduced risk of lung cancer and other types of cancers, cardiovascular diseases, and lung diseases such as chronic obstructive pulmonary disease [4]. Despite this, tobacco-use cessation is challenging and difficult to achieve without the support of a healthcare professional. Tobacco-cessation interventions offered by healthcare professionals are more effective in comparison with self-help [5]. Pharmacists practicing in community pharmacies are among the most accessible healthcare professionals by the public for providing advice on health and self-care. Evidence has proven that pharmacists have a pivotal role in health promotion and disease prevention including tobacco-cessation interventions [6, 7]. Indeed with the emergence of the pharmaceutical care concept more than two decades ago, the focus of pharmacists has shifted from product-oriented practice to patient-centered care [8]. Many pharmacists, worldwide, have started applying behavioral interventions to improve health outcomes [6]. In addition, prescription and over-the-counter pharmacotherapies for smoking cessation are readily available in community and retail pharmacies. Nicotine replacement therapy (NRT) is an example of an over-the-counter smoking cessation aid available in most countries including Qatar. Prescription smoking cessation medications include bupropion and varenicline. The availability of these medications provides pharmacists with an opportunity to help patients quit smoking and to combat tobacco use in the community [9]. Pharmacist-delivered tobacco-cessation interventions have been described in the literature [9–15]. For example, Saba et al. have conducted a meta-analysis of the effectiveness of pharmacist-delivered smoking cessation interventions within community pharmacy. The analysis has demonstrated that community pharmacy-based smoking cessation interventions can improve smoking abstinence rates [9].

The International Pharmaceutical Federation (FIP) issued a policy statement in relation to the pharmacist’s role in reducing tobacco use in the community [16]. The U.S. Department of Health and Human Service clinical practice guidelines for treating tobacco use and dependence include strategies to assist clinicians in delivering tobacco-cessation interventions [5]. Furthermore, healthcare providers, including pharmacists, were highly endorsed for delivering tobacco-cessation services in the American Society of Health-System Pharmacists’ (ASHP) Therapeutic Position Statement on the Cessation of Tobacco Use [17].

Tobacco use is a major preventable cause of mortality and morbidity in Qatar. As per the 2013 Global Adult Tobacco Survey (GATS), 12.1% of adults and 20.2% of men smoke tobacco, and 55.4% of smokers smoke an average of 16 cigarettes per day [18]. Moreover, according to the 2013 Global Youth Tobacco Survey (GYTS), 15.7% of school students in Qatar within the age range of 13 and 15 years currently use tobacco [19]. Tobacco-induced diseases including cardiovascular diseases (CVDs) are highly prevalent in Qatar. The mortality rate from CVDs from 2011 to 2013 was 8.3 per 100,000 for Qatari males aged 20–44 years [20]. Furthermore, lung cancer is one of the main causes of cancer-related mortality in males accounting for 17.4% of cancer-related deaths in 2014 [21]. Qatar residents purchase at least one billion cigarettes annually and 150 million dollars are spent per year to cover the hospital fees for smoking-related diseases [22]. To reduce the use of tobacco, Qatar has ratified the World Health Organization (WHO)’s Framework Convention on Tobacco Control (FCTC) [23] and has implemented several initiatives to reduce tobacco use including banning all forms of tobacco advertisement in media, prohibiting smoking in enclosed public areas, forbidding the sale of cigarettes to children under the age of 18 years, introducing graphic/pictorial health warnings on cigarette packs, and others [23, 24]. Despite these initiatives, tobacco use is considered a public health threat in Qatar. Qatar pharmacists and other healthcare providers have excellent opportunities to reduce tobacco use in the country. Currently, Qatar has over 1000 practicing community pharmacists and smoking cessation aids including NRT and varenicline are available over-the-counter in community pharmacies in Qatar [25]. Despite this, published studies have shown that community pharmacists in Qatar are not actively contributing to tobacco control [26]. Only 21% of community pharmacists in Qatar always or most of the time ask patients about their tobacco use or smoking status and less than 50% always or most of the time provide counseling to the purchasers of NRT. Furthermore, 89% of the community pharmacists reported that they did not receive any kind of education or training about smoking cessation counseling in the past. However, 85% of the pharmacists were interested in receiving additional training related to smoking cessation competencies/skills including initiating discussions with patients about smoking, assessing patients’ nicotine dependence, counseling patients using cognitive behavioral techniques, and other topics [26].

To be competent in providing tobacco-cessation interventions and to offer effective tobacco-cessation programs, pharmacists should have the necessary competence, skills, and confidence in relation to tobacco cessation. Several educational interventions were designed and implemented to enhance the knowledge and skills of healthcare professionals in relation to tobacco cessation [27]. Yet the majority of these interventions were implemented and assessed outside the Middle East (ME) region and did not target pharmacists. In addition, these interventions were only delivered by one group of professionals (e.g., nurses, physicians, or pharmacists) with expertise in smoking cessation programs. In an effort to build the capacity of pharmacists on tobacco-use treatment, the aim of this study is to design, implement, and evaluate an intensive education program on tobacco treatment for pharmacists in Qatar. We hypothesize that pharmacists who participate in the program will have better knowledge, attitudes, perceived self-efficacy and skills in relation to tobacco-use treatment than pharmacists who receive no such program (control group).

### Study objectives

The aim of this randomized controlled study is to design, implement, and evaluate an intensive education program provided by team of health professional educators on tobacco-use treatment for pharmacists in Qatar.

The primary objectives of the study are to assess the effectiveness of the program on pharmacists’ knowledge and skills in relation to tobacco and tobacco cessation assessed using a multiple-choice-based evaluation instrument and an Objective Structured Clinical Examination (OSCE), respectively. The secondary objectives are to assess the effectiveness of the program on pharmacists’ attitudes toward tobacco cessation and perceived self-efficacy (i.e.. confidence) in tobacco-cessation interventions, assessed using a survey instrument, and to assess the effectiveness of the program on pharmacists’ tobacco-cessation skills in the practice setting, assessed using the simulated client approach.

## Methods/design

### Study design

**Fig. 1:**
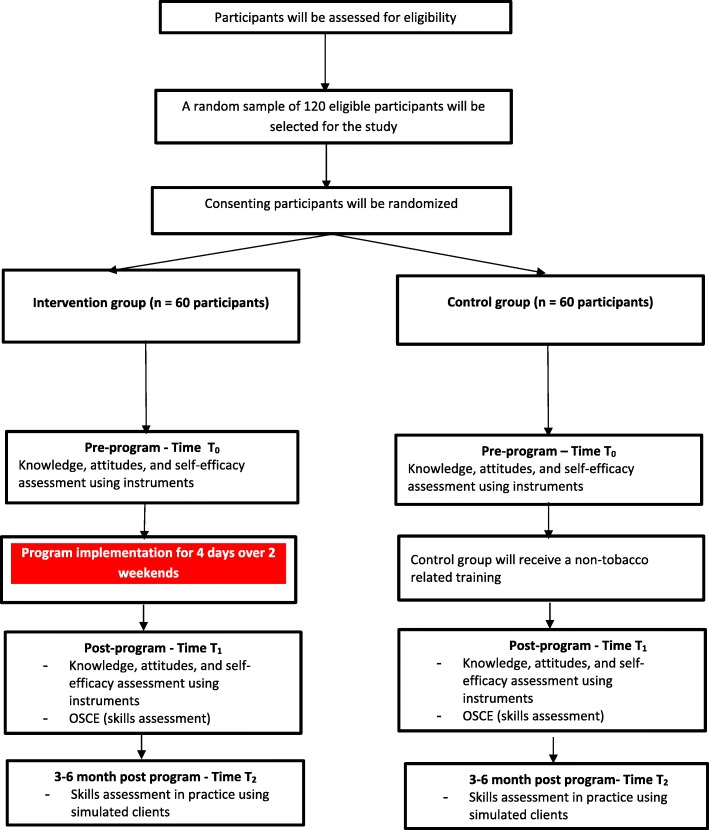
A flow chart of the study methodology and processes

**Fig. 2:**
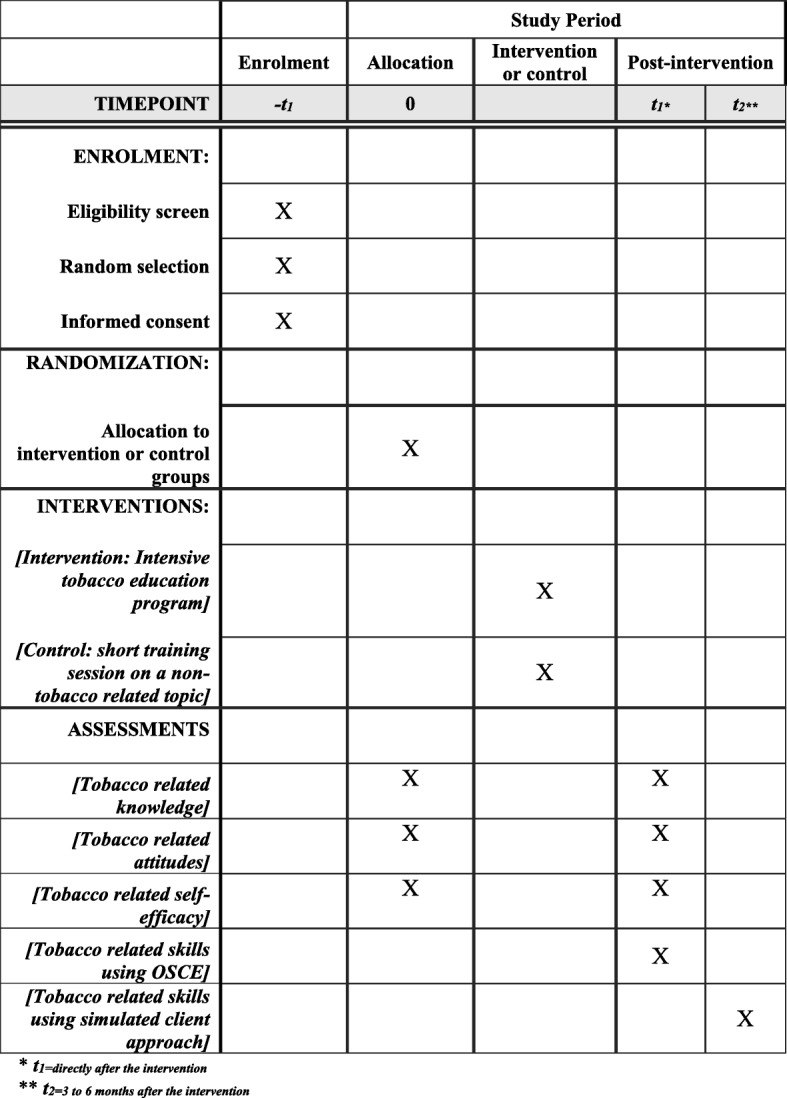
Standard Protocol Items: Recommendations for Interventional Trials (SPIRIT) diagram

The study is a prospective randomized controlled trial comparing the effectiveness of the education program, versus non-tobacco-related training, on pharmacists’ tobacco-related knowledge, attitudes, self-efficacy, and skills. The study methodology and procedures are shown in Fig. 1. The protocol is developed in accordance with the Standard Protocol Items: Recommendations for Interventional Trials guidelines (SPIRIT; Fig. 2 and Additional file 1).

The evaluation of the effectiveness of the program will be conducted using multiple strategies as follows:

- Assessment of pharmacists’ knowledge, attitudes, and self-efficacy before and after the training program
- Assessment of pharmacists’ skills using an authentic method (i.e., an OSCE) directly after the completion of the training program
- Assessment of pharmacists’ skills in the practice setting 3 to 6 months post training program using a simulated client approach

### Study-eligible participants

All community pharmacists practicing in Qatar are eligible for participation in the study. There are no specific exclusion criteria for participants’ enrollment in the study.

### Recruitment of participants and randomization

Information about the study and its purpose were made available to the Ministry of Public Health, which subsequently made available a database that has contact details of all community pharmacists practicing in the country (around 1000 pharmacists). A stratified random sample of 200 pharmacists was obtained from the list. Stratification was done by pharmacy so that no more than one pharmacist from each pharmacy would participate. Invitations to participate in the study will be made by the study research assistants (RAs) over the phone or in writing. Those who express interest will be sent a Participant Information Sheet (PIS) and a consent form. Consenting participants will be randomly allocated to intervention or control groups using permuted block randomization with blocks of size 2, 4, and 6. Randomization will be made by the study statistician who is not involved in the recruitment of the pharmacist and will be concealed for the RAs recruiting the pharmacists. Blinding of study participants in relation to their allocation to the study groups will not be possible given the nature of the intervention.

#### Intervention group

Participants in the intervention group will receive an intensive education program delivered by a group of educators, researchers, and clinicians with expertise in tobacco control and tobacco dependence treatment. The program will be delivered at Qatar University over 4 days (run over two weekends) with an average of eight contact hours per day (i.e., approximately 32 contact hours). The program will target the Core Competencies for Evidence-based Treatment of Tobacco Dependence by the Association for the Treatment of Tobacco use and Dependence (ATTUD) [28].

The training curriculum will cover the following topics:

- Tobacco-use epidemiology in Qatar and globally
- Risks and consequences of tobacco use
- Benefits of quitting tobacco use
- Principles of nicotine addiction
- Role of pharmacist in implementing tobacco-cessation interventions
- Transtheoretical model (TTM) for behavior change as applied to tobacco-use cessation
- Application of the TTM to provide tobacco cessation targeted to the patient’s readiness to change
- Behavioral modification techniques for tobacco cessation
- Use of NRT including: indications, the choice of dosage form, dosing regimen, duration of therapy, adverse effects, precautions, contraindications, tapering, pharmacokinetic and pharmacodynamic drug interactions, and patient counseling points
- Pharmacist and patient information on NRT and other cessation aids
- Use of alternative medications (varenicline, bupropion, etc.) and modalities for cessation of tobacco use
- Nicotine withdrawal symptoms
- General verbal and nonverbal communication skills
- Patient counseling and interviewing techniques including the ABC (ask, brief advice, cessation treatment) smoking cessation strategy and the 5A’s counseling process (ask, advise, assess, assist, and arrange)
- Development of a personalized action plan to assist the patient with smoking cessation
- Documentation in the patient profile
- Tobacco use and cessation in special populations (e.g., young patients, elderly, pregnant women, and others)

The program will be provided through didactic lectures and through active learning strategies including problem-based learning (PBL) exercises using case scenarios, group discussions, games, role plays, videos, simulated practical applications with peers and standardized patients, self and peer debriefing and performance feedback activities. At the end of the program, the pharmacists will be provided with a video recording of all the program content, copies of tools that they can implement in practice and information on online resources containing additional tobacco-cessation-related content. They will also be encouraged to contact the research team at any time if they have any questions or concerns. At the conclusion of the training program, all the pharmacists in the intervention group will receive continuous education (CE) units accredited by Qatar Council for Healthcare Practitioners (QCHP) of the Qatar Ministry of Public Health.

#### Control group

A short didactic session on a non-tobacco-related topic will be delivered at Qatar University to pharmacists in the control group to avoid contaminating this group with elements that could change their behavior and shift it to another level not representative of their tobacco-cessation-related “current practice” or “usual care.” Pharmacists in the control group will also receive CE units accredited by Qatar Council for Healthcare Practitioners (QCHP) of the Qatar Ministry of Public Health. Examples of potential topics that will be covered in the control group are in relation to women’s health such as contraception.

### Outcome measures

The study has two primary outcomes: post-intervention tobacco-related knowledge and post-intervention skills for tobacco cessation assessed using a multiple-choice-based evaluation instrument and an OSCE, respectively. The secondary study outcomes are post-intervention attitudes towards tobacco cessation and self-efficacy in tobacco-cessation interventions assessed using a survey instrument. An additional secondary study outcome is the post-intervention performance difference in relation to tobacco-cessation skills in the practice setting assessed using the simulated client approach.

### Pre- and post-program assessment of pharmacists’ knowledge, attitudes, and self-efficacy

#### Study procedures

This evaluation of the program effectiveness will utilize a before-and-after design between the two groups. The 4-day education program for tobacco treatment will serve as the intervention. Pre-tested instruments will be used to assess knowledge, attitudes and perceived self-efficacy (i.e., confidence) among intervention participants and control participants before and after the program.

#### Setting

This evaluation will be conducted in the same venue as the training program (i.e., Qatar University).

#### Assessment implementation

The assessment instruments will be distributed to participants in both groups prior to, and following, the program. The assessment will be likely online using a survey software. Participants will be allowed a certain predetermined time to complete the evaluations. Unique participants’ codes will be created to link the pre to post data for the same participant.

### Post-program assessment of pharmacists’ skills using the OSCE

#### Design and structure of the OSCE

Effective provision of tobacco-cessation intervention requires significant amount of skills in addition to the relevant knowledge. The proposed program has been carefully designed to cover a set of skills and competencies. Assessing participants’ practical skills using the traditional assessment methods (e.g., multiple choice questions (MCQs) or short essay) may not be appropriate in assessing skills associated with tobacco cessation An alternative means to assess a pharmacist’s practical skills in the delivery of effective tobacco cessation is the use of an authentic, performance-based assessments, such as the OSCE. Examples of skills to be assessed include communication skills in general, counseling skills, professional practice and ethics, and interviewing skills. We propose a five- to eight-station OSCE that will target core competencies and skills covered in the program. Participants in both groups will do the OSCE. In the OSCE, each participant will be allocated 10 min to interact with a standardized patient (SP) who will be trained using a validated script. Performance of participants will be assessed using validated assessment checklists. The cases and the assessment checklists will be reviewed for content and face validity by at least two faculty members who have expertise in tobacco cessation and suggested changes will be incorporated. Assessors for each station will be trained on using the checklists and will be blinded in relation to the study participants’ groups. The OSCE will be conducted in the same venue as the training program.

### Three- to six-month post-program assessment of pharmacists’ skills using simulated client approach

In this phase, the study will evaluate whether participants in the intervention and control groups will offer appropriate tobacco-cessation counseling and will recommend proper tobacco-cessation aids in their practice setting. Using a simulated client approach to data collection 3 to 6 months post training program, the study will assess the appropriateness of advice and recommendations given to a simulated client who asks for assistance in quitting smoking. The simulated client method is recognized and used internationally for assessing the quality of services offered by pharmacists including smoking cessation [29–32]. Simulated clients using two designed and validated case scenarios will visit participants in both groups. The clients will be selected to resemble the sociodemographic and practice characteristics of the Qatar population and its residents. Objective assessment forms will be developed using tobacco-cessation guidelines including the U.S. Department of Health and Human Service clinical practice guidelines (CPGs) for treating tobacco use and dependence and the Qatar guidelines for management of tobacco dependence as guides [5, 33]. Individual items in assessment forms will be completed using dichotomous scales (closed-ended yes/no questions) and will target different aspects of pharmacist performance including gathering information and patient history-taking, assessing the patient, recommending an appropriate treatment plan, and providing counseling. Research assistants will accompany the simulated clients in their visits. They will complete the assessment forms and will record the notes summarizing the encounter. One-to-one training will be provided to simulated clients and RAs by the study research team. Training will offer opportunities for the shoppers to role-play and to become proficient in their assigned scenarios, will help in refining the assessment sheets, in standardizing the process for all participants and in decreasing inter-rater variability. Both simulated clients and RAs will be blinded for the pharmacist group allocation. Each simulated client will enter the community pharmacy and will ask to speak to the pharmacist. The client will start with designed queries as per an assigned scenario. The pharmacist will then be given the chance to offer the counseling. After the visit is completed, an explanation of the purpose of the visit and how the collected data will be de-identified will be shared with the pharmacists. Anonymity and confidentiality will be ensured. Consent to participate in this phase of the study will be sought at this time. For pharmacists who decline to participate, information collected during the patient encounter will be discarded.

### Sample size estimates

With 54 pharmacists per group, the study will have an 80% power to detect an effect size of 0.60 between the study groups for two primary outcomes which are numeric (knowledge and skills scales) using the independent *t* test with a significance level of 0.025%. In addition, with 54 pharmacists per group, the study will be able to detect a difference of at least 27.5% between the two study groups for any dichotomous outcome using the chi-square test with 80% power and a significance level of 5%. Assuming a loss-to-follow-up rate of 10%, the study will randomize 60 pharmacists per group for a total of 120 pharmacists. With over 1000 community pharmacists practicing in Qatar, including 60 pharmacists in each group is achievable. We will select a random sample of 200 pharmacists for recruitment as we anticipate that some pharmacists will not accept to participate. Once the sample size of 120 is reached the recruitment will stop.

### Statistical analysis

Data will be analyzed using IBM SPSS (IBM SPSS® for Windows, Version 24.0; IBM Corp, Armonk, NY, USA). The Consolidated Standards of Reporting Trials (CONSORT) guidelines will be followed when analyzing the study data [34]. Demographic and other pharmacist education and practice-related questions, along with baseline knowledge, attitudes, and perceived self-efficacy, will be summarized using means and standard deviations for numeric variables such as age and year of experience and frequency distribution for categorical variables such as gender, country of education, etc. Those will be compared between the study groups so that differences will be used to adjust the primary analyses.

The primary analyses will include comparing post-knowledge scores between the two study arms using the independent *t* test and the skills-related scores on the OSCE using the same method. The secondary analyses will include adjusting the main analyses to any imbalances in baseline variables that are observed between the study arms. This will be done using the linear regression method. Moreover, knowledge scores will be compared within each study group using the paired *t* test. For outcomes that are dichotomous or that will be dichotomized, such as the percentage of pharmacists with a specific attitude or self-perceived efficacy, then the chi-squared test will be used to compare such outcomes between study groups and the McNemar test will be used to compare responses within each study group over time. Performance on the simulated patient will be compared in a similar manner as the outcomes above depending on whether it is dichotomized or not. In addition, repeated-measures analysis (depending on the scale of measurement for each outcome) may be used to detect whether there is an intervention × time interaction so that difference between study groups is changing over time or not. The level of statistical significance will be set at 5%.

### Ethical considerations and data handling

The study protocol and all related instruments and forms were granted ethical approval by the Qatar University (QU) Institutional Review Board (IRB) (QU-IRB 906-E/18). All data related to the study will be kept confidential and will be retained in a password-protected database maintained along with all related study documentation in a locked cabinet at QU College of Pharmacy. Participation in the study will not cause any harm to participants and, as a result, no data monitoring committee will be assigned. Only the study team will have access to the final study dataset. Any protocol modifications will be communicated with QU IRB. The study results will be presented via poster or oral presentations in international conferences and will be published in peer-reviewed journals. The authors of the final study manuscript will have considerable contributions to the design, implementation, assessment, and reporting of the study.

### Monitoring

The study progress will be monitored closely by the study team. Meetings will be held to guide the study towards its aim and objectives with team involvement. A weekly meeting involving all the team members including RAs will be organized. The study team will be in direct touch with each other and RAs through electronic communication (Google® docs and emails). Electronic communication will help in keeping the minutes of the meetings, will provide easy access to any study-related document, and will facilitate online sharing of information. The study team will also conduct ad-hoc meetings at least biweekly to monitor data collection, to check protocol adherence, to review appropriate documentation and to address any issues that might arise.

## Discussion

Many published studies have demonstrated the impact of tobacco-cessation-related training on pharmacists’ knowledge, self-efficacy, confidence, and ability to provide tobacco-cessation counseling [35]. This study will be expected to be the first randomized controlled trial conducted within Qatar and the ME that includes designing, implementing, and evaluating an intensive education program on tobacco-cessation treatment for pharmacists in Qatar. The findings of this study should have far-reaching implications to add to the existing body of knowledge about the management of what is known to be an endemic health hazard costing countries billions of dollars every year and negatively affecting the quality of life of millions of people and their families. In response to the health effects of smoking and its spread, the WHO introduced strategies to combat smoking through the implementation of its Framework Convention on Tobacco Control (FCTC) entitled MPOWER. The six measures under this strategy include monitoring tobacco use and prevention policies; protecting people from tobacco use; offering help to quit tobacco use; warning about the dangers of tobacco; enforcing bans on tobacco advertising, promotion, and sponsorship; and raising taxes on tobacco [1]. Several measures under the “offering help to quit tobacco use” strategy require skilled and trained personnel. The role of pharmacists in helping patients quit smoking is well established. Pharmacists could use their skills and training to become providers of a credible source of health information [9]. However, the skills to help people change their unhealthy behaviors are not generic. The proposed program aims at providing specialist-level training for generalist pharmacists to provide them with the skills and knowledge tailored to assist people who smoke to quit smoking.

Tobacco cessation is one of the most cost-effective of all healthcare interventions and a successful program within the healthcare system will release resources for other needs. The FCTC contains specific obligations concerning tobacco dependence and cessation, including the establishment of programs for diagnosing, counseling, preventing, and treating tobacco dependence [36]. This study builds the foundation for a new form of collaborative interprofessional healthcare team to confront the endemic nature of smoking. The training team consists of expertise from academia and healthcare organizations in the three countries: Qatar, New Zealand, and Malaysia. The training program is anticipated to generate individual healthcare practitioners with the right set of skills and competence to join the current teams at the Tobacco Control Center-WHO Collaborating Center at Hamad Medical Corporation in its efforts within a unique professional approach. This study has the potential to create the right environment for a scheme of continued and seamless smoking cessation care in which referral of patients between primary care and Hamad Medical Corporation (HMC: the main public hospital in Qatar) can be facilitated. The experience and information generated by this study in these aspects should be of interest at the national, regional, and international level.

## Trial status

Preparation for the training program is currently ongoing. It is expected that the recruitment of participants will be held in August 2018 and that the program will be delivered in September 2018.

## Additional file

Additional file 1: Standard Protocol Items: Recommendations for Interventional Trials (SPIRIT) 2013 Checklist: recommended items to address in a clinical trial protocol and related documents*. (DOC 139 kb)

## Abbreviations

ASHP: American Society of Health-System Pharmacists
ATTUD: Association for the Treatment of Tobacco use and Dependence
CE: Continuous education
CPG: Clinical practice guidelines
FCTC: Framework Convention on Tobacco Control
FIP: International Pharmaceutical Federation
GATS: Global Adult Tobacco Survey
GYTS: Global Youth Tobacco Survey
HMC: Hamad Medical Corporation
MCQ: Multiple choice question
ME: Middle East
NRT: Nicotine replacement therapy
OSCE: Objective Structured Clinical Examination
PBL: Problem-based learning
QCHP: Qatar Council for Healthcare Practitioners
QU: Qatar University
SPIRIT: Standard Protocol Items: Recommendations for Interventional Trials
SPSS: Statistical Package of Social Sciences
TTM: Transtheoretical model
WHO: World Health Organization
